# Modeling Outcomes of First-Line Antiretroviral Therapy and Rate of CD4 Counts Change among a Cohort of HIV/AIDS Patients in Ethiopia: A Retrospective Cohort Study

**DOI:** 10.1371/journal.pone.0168323

**Published:** 2016-12-20

**Authors:** Tadesse Awoke, Alemayehu Worku, Yigzaw Kebede, Adetayo Kasim, Belay Birlie, Roel Braekers, Khangelani Zuma, Ziv Shkedy

**Affiliations:** 1 Epidemiology and Biostatistics, University of Gondar, Gondar, Ethiopia; 2 School of Public Health, Addis Ababa University, Addis Ababa, Ethiopia; 3 Wolfson Research Institute, Durham University, Durham, United Kingdom; 4 Biostatistics, Jimma University, Jimma, Ethiopia; 5 I-BioStat, Hasselt University, Diepenbeek, Belgium; 6 Health Sciences Research Council, Pretoria, South Africa; Fundacao Oswaldo Cruz, BRAZIL

## Abstract

**Background:**

Antiretroviral therapy has shown to be effective in reducing morbidity and mortality in patients infected with HIV for the past couples of decades. However, there remains a need to better understand the characteristics of long-term treatment outcomes in resource poor settings. The main aim of this study was to determine and compare the long-term response of patients on nevirapine and efavirenz based first line antiretroviral therapy regimen in Ethiopia.

**Methods:**

Hospital based retrospective cohort study was conducted from January 2009 to December 2013 at University hospital located in Northwest Ethiopia. Human subject research approval for this study was received from University of Gondar Research Ethics Committee and the medical director of the hospital. Cox-proportional hazards model was used to assess the effect of baseline covariates on composite outcome and a semi-parametric mixed effect model was used to investigate CD4 counts response to treatments.

**Results:**

A total of 2386 HIV/AIDS naive patients were included in this study. Nearly one-in-four patients experienced the events, of which death, lost to follow up, treatment substitution and discontinuation of Non-Nucleoside Reverse Transcriptase Inhibitors(NNRTI) accounted: 99 (26.8%), 122 (33.0%), 137 (37.0%) and 12 (3.2%), respectively. The hazard of composite outcome on nevirapine compared with efavirenz was 1.02(95%CI: 0.52-1.99) with p-value = 0.96. Similarly, the hazard of composite outcome on tenofovir and stavudine compared with zidovudine were 1.87 (95%CI: 1.52-2.32), p-value < 0.0001 and 1.72(95% CI: 1.22-2.32), p-value = 0.002, respectively. The rate of CD4 increase in response to treatment was high during the first 10 months and stabilized later.

**Conclusions:**

This study revealed that treatment responses were comparable whether nevirapine or efavirenz was chosen to initiate antiretroviral therapy for HIV/AIDS patients in Ethiopia. There was significant difference on risk of composite outcome between patients who were initiated with Tenofovir containing ART regimen compared with zidovudine after controlling for NNRTI drug combinations.

## Introduction

The scale-up of antiretroviral therapy (ART) for HIV/AIDS patients in resource-limited settings has been one of the largest public health operations in many African countries, and by the end of 2013 more than 11.7 million people were receiving ART in low and middle-income countries [1, 2]. ART has been shown to be effective in reducing morbidity and mortality in patients infected with HIV [3, 4]. It averts 5.5 million deaths in low and middle income countries from the peak in 1995 until 2013. Sub-Saharan Africa accounted for most of those lives [5]. The goal of ART is to attain maximal and durable suppression of the viral replication and prolong diseases free survival [6].

The revision of the World Health Organization (WHO) HIV-treatment guidelines in 2010 brought several changes to the management of HIV-infected patients [7]. Among them was a statement about progressing to less toxic antiretroviral drugs in first-line regimens. Since 2006, WHO has recommended in its HIV/AIDS treatment guidelines that treatment providers begin moving away from the drug Stavudine (d4t) because of its long-term irreversible side effects, and instead to use either Tenofovir (TDF) or zidovudine (AZT) as Nucleotide Reverse Transcriptase Inhibitor (NRTI) backbone [8]. European Medicines Agency recommended that, in view of its long-term toxicities, d4t be used for as short time as possible and only when no appropriate alternatives exist [9].

The effectiveness of ART has been assessed by clinical observations, CD4 cell counts and determination of plasma viral load [10]. The Non-Nucleoside Reverse Transcriptase Inhibitors (NNRTIs) are drugs choices for initial ART for HIV infection. Studies in resource-rich settings revealed that efavirenz (EFV) containing regimen has better treatment outcomes than nevirapine (NVP) containing regimen [11, 12]. A randomized clinical trial in India [13] also showed that regimen containing nevirapine (NVP) was inferior and was associated with more frequent virologic failure and death. This was supported in some resource-poor settings including Swaziland, Zambia and Botswana as well [14–16]. However, there exist evidence in resource-poor settings that shows as there was no difference between EFV and NVP in the long-run [17–19].

In the national treatment guideline of Ethiopia 2010, the first-line ART contains four NRTIs backbone (Stavudine (d4t), zidovudine (AZT), Abacavir (ABC) and Tenofovir (TDF)) plus lamivudine (3TC) and two NNRTI drugs (efavirenz (EFV) or nevirapine (NVP)) [10]. The combination regimens which have been used most frequently in Ethiopia are d4t-3TC-EFV, d4t-3TC-NVP, AZT-3TC-EFV, AZT-3TC-NVP, TDF-3TC-EFV, or TDF-3TC-NVP. When the patient is unable to tolerate the side-effect due to toxicity, the offending drug can be substitute with another drug that does not have the same side-effect [20]. Whereas, patients switch to second-line regimen when the first-line regimen failed due to different reasons. A failure in treatment is measured in three ways: (1) clinical-when new or recurrent WHO stage 4 condition, (2) immunological-when persistent CD4 level below 100 or 50% fall from on-treatment peak value and (3) virological-when plasma viral load above 10,000 copies/ml in duplicates after six months on ART [10].

The choice of treatment combinations for HIV/AIDS patients to initiate ART depends on cost and efficacy. Thus, knowing the long-term treatment outcomes of more costly drugs is very decisive for decision making in resource limited nations. In this study we aim to determine the long-term outcomes of first-line ART drugs and rate of change in log-CD4 cell counts in response to antiretroviral treatments. Furthermore, the effect of treatment choices at the initiation on CD4 evolution was compared and tested as well. This paper is organized as follows. In Section 2, we present the methods. In Section 3, present the result of survival and longitudinal models. In Section 4, the finding of the two approaches were discussed and concluding remarks were given.

## Methods

### Study Population

Gondar University hospital ART clinic started treating HIV/AIDS patients as part of the National AIDS control program since 2005. At the same time ART was started to be provided for free in the selected hospitals in the country, Gondar University hospital is one of these hospitals. As a result, patients were referred to Gondar University hospital from many areas in Northwest Ethiopia.

The study included ART naïve patients aged ≥ 15 years-old who initiated ART containing TDF, d4t, or AZT as NRTI backbone with NVP and EFV as NNRTI drugs between 2009 and 2013. In total, following the inclusion criteria, 2386 patients included in this study. Data on patients were recorded in patients’ chart and entered into access database which was designed for this purpose. Baseline characteristics such as sex, age, weight, WHO staging and functional status were collected when the patient enrolled in the clinic. Whilst clinical variables such as CD4 cell counts, and regimen were collected every 6 months subsequently depending on the progress of the patient. In some cases, patient’s visit might be taken at irregular time due to different reasons such as diseases progression, toxicity or opportunistic infections. The criteria for initiating ART in Ethiopia followed WHO guideline [7], with an adjustment of CD4 threshold from 200 to 350 *cell*/*mm*^3^ in 2010.

### Data and Study Variables

Data for the study were accessed from ART clinic database which had been collected from HIV/AIDS patients who initiated ART from January 2009 to December 2013. As mentioned above, data were recorded in the patients’ chart by health professional at enrollment and at each visit. Since 2009 the hospital used electronic recording system (database) prepared for this purpose. The information collected by the health professional from the patient were sent to the data manager who entered the data into the computer. Information on treatment substitution, treatment discontinuations, death, lost to follow up and transferred out were obtained from hospital records.

For this study, *NNRTI substitution, treatment discontinuation, transferred out, lost to follow up and death were defined as follows*: NNRTI substitution is modifying NNRTI drugs of the original regimen for any reason. *treatment discontinuation* is when the patient changed his/her first-line regimen to second-line regimen. *Lost to follow up* was defined as missing a clinic appointment for more than three months without further attendance at clinic. *Transferred out* was defined as transfer of patients to other ART clinic with all the history/records. *Death* was defined as confirmed deaths from medical records or verbal confirmation of death by relatives or friends. Patients who had at least two CD4 counts (two visits for those who experienced the event) were included for the study. Information on baseline characteristics were obtained from registry in the database. Whereas,follow-up variables were accessed from ART refill. The data were closed for analysis on December 31, 2013.

#### Data quality Control

The ART clinic of Gondar University hospital has been using database to enter information of the patients starting from the first visit. It was developed by information technologist and well tested before used for data entry in the hospital. Well trained data entry clerks employed by the hospital perform the data enter. The entry process is supervised by the data manager for completeness and consistency in daily bases. The data for this study was accessed from this database. When data on important variable (CD4 counts, regimen, and last patient status), is missed in the database, it was retrieved from patients’ chart at the chart room.

### Data Analysis Methods

#### Data analysis for time to event outcomes

We compared patient characteristics at ART initiation by initial NNRTI treatment groups (EFV or NVP) using chi-square test for categorical covariates and Wilcoxon rank sum test for continuous covariates. There were different responses to ART treatment. A composite endpoint which represents different responses to ART treatment was defined and analyzed as time to event endpoint. These include drug substitution, lost to follow up, treatment discontinuation, and death. Three types of survival analysis were considered; primary analysis (NNRTI substitution and lost to follow up were treated as censored), two sensitivity analysis (NNRTI substitution and/or lost to follow up were treated as event).

For the primary outcome, time to the first occurrence of any of the outcome measures was calculated by subtracting the date of the event from the date of initiation of ART. Patients were censored if death was not observed until the time of the last visit for patients who were lost and December 31, 2013 for patients who were alive. Note that we assessed NNRTI substitution and discontinuation as an event [21]. Discontinuation of NNRTI was defined as discontinuation of either NVP or EFV due to toxicity, or patient or physician preference.

Log-rank test was used to compare between groups of baseline categorical variables. Cox-regression analysis was used to identify predictors for the time to event outcomes. Baseline covariates such as sex, age (≥ 40 versus <40 years), WHO clinical stage (IV or II versus I or II), CD4 cell counts (<200 versus ≥200), calendar year (before 2010 versus since 2010), NRTI backbones (d4t plus 3TC, AZT plus 3TC or TDF plus 3TC) and NNRTI drugs (EFV versus NVP) were considered. The categorization of numeric variables was done based on other previous studies [21] [22] for comparison purpose. Similarly, Cox-regression analysis was used to compare the baseline covariates for their risk of composite outcome [23]. The hazard ratio with 95% confidence interval was used to test statistical significant association. A detail discussion about the cox-regression can be referred in the supporting information section (S1 Appendix).

#### Data analysis for Immunological outcomes

Treatment effects on the CD4 cell counts evolution varies over time and it is expected that repeated measurements taken on the same subject to be correlated. A linear mixed-effect models [24] can be used to account for this types of correlations [25]. However, the trajectory of CD4 cell counts over time is not purely linear, but nonlinear. To investigate the effect of treatment on the change in CD4 cell counts over time, we used Semi-parametric mixed effects model [26] which is a data driven approach. Such a model can capture both linear and non linear trend in the data. It allows smoothing with respect to *time*. For this model, log transformed CD4 cell counts over time was fitted using the *R* package mgcv. Furthermore, the first order derivatives for each treatment groups were plotted with 95% confidence band in order to determine the effect of treatments on the rate of change in log CD4 cell counts over time. An elaborate discussion about the semi-parametric mixed model is given in the supporting information section (S1 Appendix).

### Ethical Clearance

A human subject research approval for this study was received from Institutional Review Board (IRB) of the University of Gondar. As the study was retrospective, the IRB waived that the research could be done based on record review without contacting the patients. Support letter was obtained from the medical director office of the hospital for retrieving retrospective data from the database and records. All the information was kept confidential, and no individual identifiers were collected.

## Results

### Baseline Description

Majority of the patients, 1462 (61.27%) were initiated with NVP containing Non-Nucleoside Reverse Transcriptase Inhibitors (NNRTI); of whom 1023 (70.0%) used AZT as Nucleoside Reverse Transcriptase Inhibitors (NRTI) backbones. Patients who were initiated with treatment containing NVP were predominantly female 927 (63.41%) and were younger than 40 years were 1132 (77.43%). Among patients who were initiated with EFV containing treatment, 140 (15.2%) were ambulatory as compared to 124 (8.5%) who initiated with NVP containing treatment. At initiation most patients, 1149 (48.2%) were at clinical stage III, of which 483 (52.3%) and 666 (45.5%) were initiated with EFV and NVP containing treatments respectively. The median CD4 cell count was higher for those who were initiated with NVP as compared to EFV (152 versus 141); however significant difference was not found with regard to age and weight among the two treatment groups (Table 1). There was no significant difference between EFV and NVP with regard to baseline CD4 cell counts, but association was observed with other baseline covariates.

**Table 1:** Cohort characteristics at initiation of ART by Non-nucleotide Reverse Transcriptase Inhibitor (NNRTI) of HIV/AIDS patients in Gondar University Hospital, Northwest Ethiopia, 2013.

Characteristic	Efavirenz, n = 924	Nevirapine, n = 1462	P-value
**Sex, n(%)**			
Female	497(53.8)	927(63.4)	<0.0001
Male	427(26.2)	535(36.6)	
NRTI backbone, n(%)			
Zidovudine + lamivudine	215(23.3)	1,023(70.0)	<0.0001
Tenofovir + lamivudine	657(71.1)	331(22.6)	
Stavudine + lamivudine	52(5.6)	108(7.4)	
Functional Status, n(%)			
Ambulatory	140(15.2)	124(8.5)	<0.0001
Bedridden	62(4.2)	24(1.6)	
Working	722(78.1)	1,314(89.9)	
WHO stage, n(%)			
I	119(12.9)	390(26.7)	<0.0001
II	101(10.9)	285(19.5)	
III	483(52.3)	666(45.5)	
IV	221(23.9)	121(8.3)	
ART Start Year, n(%)			
2009	247(26.7)	437(29.9)	<0.0001
2010	190(20.6)	321(22.0)	
2011	154(16.7)	285(19.5)	
2012	128(13.8)	253(17.3)	
2013	205(22.2)	166(11.3)	
Age, median (IQR)	33(40-27)	31(38-27)	0.004
CD4 counts, median(IQR)	141(231-66)	152(210-84)	0.196
Weight (kg), median (IQR)	49(55-43)	50(58-45)	0.00017

### Description of composite treatment outcomes

**Fig 1:**
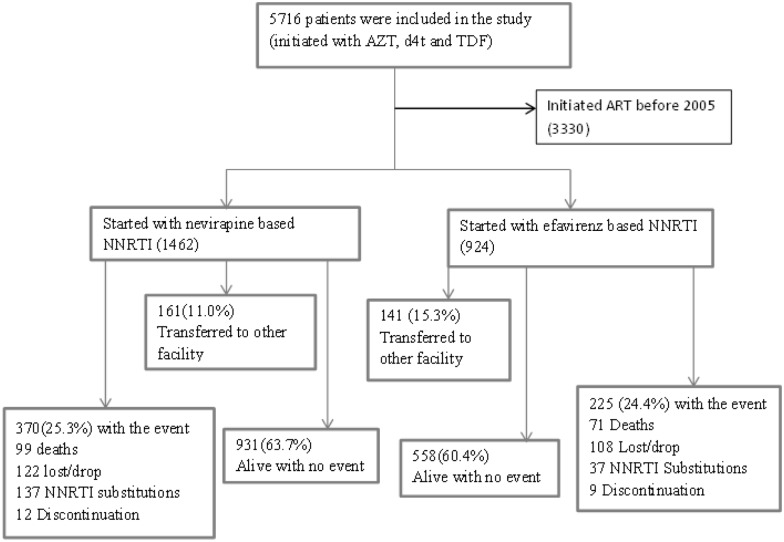
Schematic presentation showing study participants with their treatment outcomes assessed from January 2009 to December 2013.

The composite outcome was observed among 595(24.9%) patients with rate per 100 person years of 12 (95%CI: 11.1-13.0). Amongst those who were initiated with NVP 370(25.3%) patients experienced the events. Of these death, lost to follow up, NNRTI substitution, and discontinuation accounted for 99(26.8%), 122(33.0%), 137(37.0%), and 12(3.2%), respectively. Whiles among those who were initiated with EFV 225(24.4%) experienced the event. Of these death, lost to follow up, NNRTI substitution, and discontinuation accounted for 71(7.7%), 108(11.07%), 37(4.0%) and 9(0.97%), respectively. A total of 818 (55.6%) and 515(55.7%) patients stayed in their original regimen when initiated with NVP and EFV containing treatments respectively. One-in-four patients who were initiated with NVP as NNRTI drug experienced the event during the follow up period (Fig 1).

### Analysis of time to composite treatment outcomes

**Fig 2:**
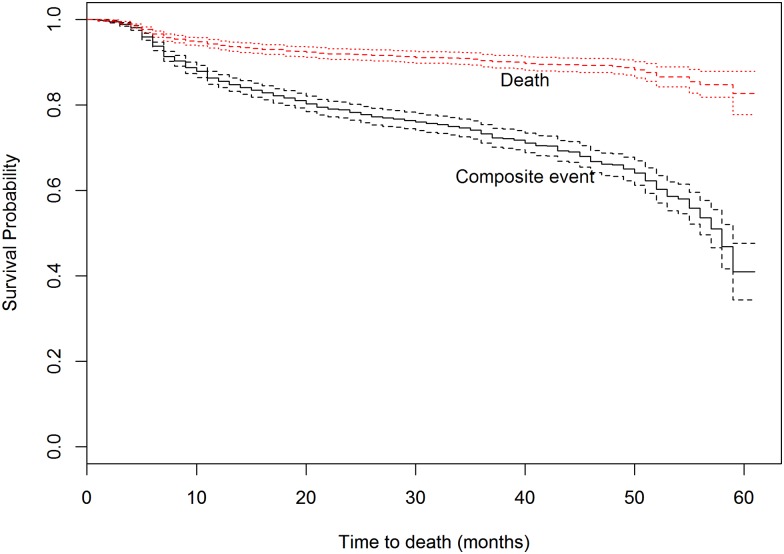
Kaplan-Meier survival curves for death and composite outcome among HIV/AIDS patients at Gondar University Hospital, 2013.

The cohort was followed for a maximum of 61 months. The cumulative probability of staying 59 months was 82.7% and contributed a total of 4958.75 person-years of the data with mean follow-up time of 25 (sd = 17.8) months. The rate of composite outcome was high during the first 10 months after ART initiation. The Kaplan-Meier survival curve for composite outcome increased sharply after 40 months. Whereas the curve for death shows steady increase (Fig 2).

**Fig 3:**
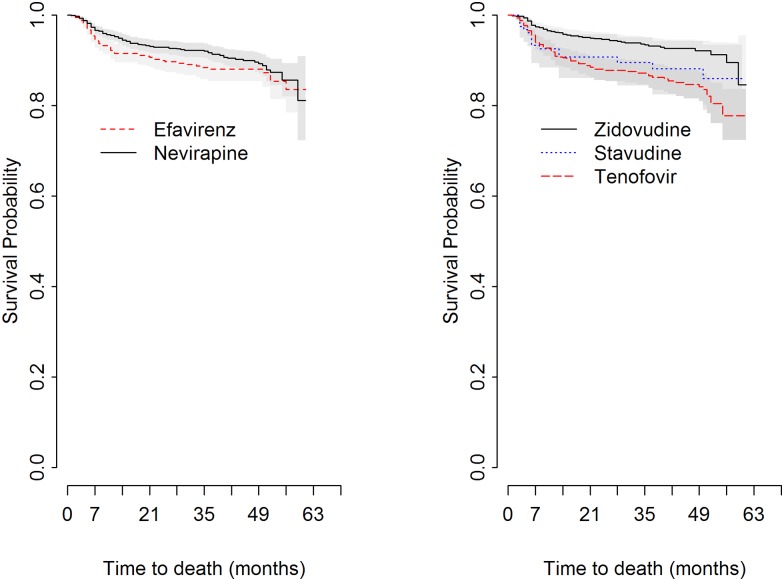
Kaplan-Meier survival plots. The proportion of surviving was plotted at anytime for NNRTI and NRTI groups at initiation and compared by log-rank test among HIV/AIDS patients at Gondar University Hospital, 2013.

Log-rank test was used to test the difference between the categories of baseline covariates with the probability of death. This test revealed the presence of significant difference among the categories of baseline NNRTI, and NRTI drugs (Fig 3). The plots of other baseline covariates were presented in S1 and S2 Figs.

The patients could experience more than one event during follow-up, and time to the first event was used for analysis. In the primary analysis, only death was considered as an event of interest. The risk of death was not different among patients on NVP compared with EFV (AHR = 1.02 (95%CI: 0.81-1.58)) which was also observed in the log-rank test. Other baseline covariates considered in the Cox-regression analysis were sex, age, NRTI backbone, WHO staging, baseline CD4 cell counts, functional status and ART start years. In the adjusted analysis, patients who were initiated with ART at old age (greater than 40 years) (HR = 1.65, 95%CI: 1.21-2.31), TDF backbone as compared to AZT (HR = 1.90, 95%CI: 1.35-2.67) and WHO stage IV or III as compared to stage II or I (HR = 1.77, 95%CI: 1.22-2.56) had higher risk of death. Whilst CD4 cell counts higher than 200 *cell*/*mm*^3^ (HR = 0.40, 95%CI: 0.25-0.64) and functional status of working (HR = 0.51, 95%CI = 0.37-0.71) had reduced risk of death as compared to ambulatory or bedridden (Table 2).

**Table 2:** Cox-regression analysis of factors associated with the composite outcome of treatment failure on first-line ART in Northwest Ethiopia, 2013.

Covariate	Primary analysis (lost as censored)			Sensitivity analysis(lost as event)		
	UHR(95%CI)	AHR(95%CI)	p-value	UHR(95%CI)	AHR(95%CI)	p-value
**Sex, n(%)**						
Female	1	1		1	1	
Male	1.18(0.88-1.57)	0.97(0.72-1.31)	0.85	1.39(1.25-1.68)	1.30(1.07-1.58)	0.007
**Age**						
<40 years	1	1		1		
≥40 years	1.66(1.23-2.24)	1.65(1.21-1.31)	0.001	1.12(0.90-1.39)	1.02(0.81-1.27)	0.88
**NNRTI**						
Efavirenz	1	1		1	1	
Nevirapine	0.75(0.56-0.99)	1.19(0.85-1.65)	0.29	0.65(0.54-0.79)	1.02(0.81-1.58)	0.88
**NRTI backbone**						
Zidovudine	1	1		1	1	
Stavudine	1.71(0.99-2.95)	1.20(0.68-2.12)	0.52	2.21(1.58-3.10)	1.73(1.22-2.46)	0.002
Tenofovir	2.20(1.62-2.98)	1.90(1.35-2.67)	.0001	2.19(1.78-2.69)	1.89(1.50-2.38)	<.001
**WHO stage**						
I and II	1	1		1	1	
III and IV	2.30(1.62-3.27)	1.77(1.22-2.56)	.0002	1.81(1.45-2.26)	1.37(1.08-1.74)	0.008
**Base CD4 cells**						
<200 cells/mm3	1	1		1	1	
≥200 cells/mm3	0.33(0.21-0.52)	0.40(0.25-0.64)	.0001	0.67(0.52-0.85)	0.79(0.62-1.01)	0.05
**Functional status**						
Ambulatory/Bedridden	1	1		1	1	
Working	0.35(0.26-0.48)	0.51(0.37-0.71)	<.01	0.40(0.32-0.49)	0.53(0.42-0.66)	<.001
**ART start Year**						
Before 2010	1	1		1	1	
Since 2010	0.67(0.50-0.91)	0.88(0.65-1.21)	0.44	0.81(0.65-0.98)	1.01(0.82-1.25)	0.93

At baseline, patients who were lost to follow up had similar CD4 counts, age, WHO stage and functional status as dead patients. Moreover, study showed that lost to follow up patients were often found to be dead when tracked [27]. Thus, sensitivity analysis was done by considering lost to follow up as event. Despite few changes, the effects of these covariates were similar with the primary analysis. However, sex and d4t backbone were significant in the sensitivity analysis, but not in the primary analysis. The risk of composite outcome was 1.30(95%CI: 1.07-1.58) times higher among males patients than the female patients. Whilst the hazard of composite outcome was 1.73(95%CI: 1.22-2.46) and 1.89(95%CI: 1.50-2.38) times higher for patients who initiated with d4t and TDF containing ART regimen respectively compared with AZT.

Characteristics at baseline might be different for patients initiated with NVP or EFV containing regimens. A secondary analysis was performed using a propensity score weighting of the model. Covariates NRTI, age, gender, baseline functional status, WHO stage, and CD4 cell counts were included in the propensity model. The continuous measure of propensity score was used as an additional covariate in the final model. The results revealed that the risk of death or composite outcome are not different among the NNRTI drugs which is consistent with multivariate Cox-regression.

#### Comparison of Efavirenz versus Nevirapine on NRTI backbone

In order to compare the risk of composite outcome on the two NNRTI drugs a Cox PH model was fitted adjusting for NRTI backbones. On a backbone of d4t and AZT, there were no significant differences in the risk of composite outcome between NVP and EFV after adjusting for other baseline covariates (sex, WHO stage, functional status and ART start year). In a similar analysis for TDF, the risk of composite outcome was 1.51(95%CI: 1.01-2.28) times higher on NVP as compared to EFV.

#### Comparison of Zidovudine, Stavudine and Tenofovir on NNRTI drugs

Similarly, NRTI backbones were compared using a Cox PH model adjusting for NNRTI drugs. In EFV of an NNRTI option, the risk of composite outcomes on TDF and D4T were 1.83(95%CI: 1.36-2.47), and 1.70(95%CI: 1.11-2.61) respectively. Likewise patients who initiated with NVP containing regimen, the hazard of composite outcomes on TDF and D4T were 2.09(95%CI: 1.38-3.18), and 0.97(95%CI: 0.46-2.04) respectively. Thus, there were significant differences in the risk of composite outcomes on TDF as compared to AZT in both NNRTI drugs.

#### Longitudinal modeling of CD4 cell counts evolution

**Fig 4:**
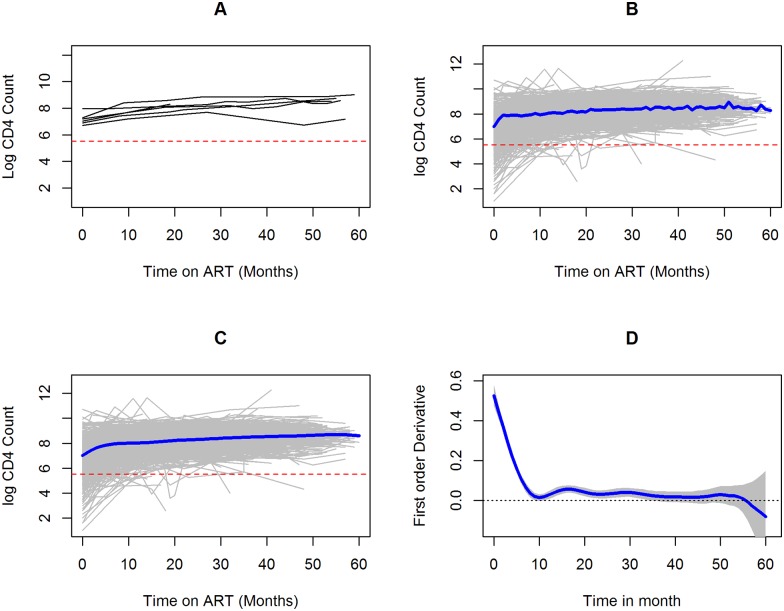
Log CD4 counts over time. (A) Individual log CD4 count trajectories of 5 selected patients, (B) Individual log cd4 count trajectories with observed average plot, (C) individual log cd4 count trajectories with predicted CD4 count trend (by the semi-parametric mixed effects model) and (D) the estimated rate of change (the first derivative) over time.

The cohort was followed for a maximum of 61 months. The median number of repeated measurements was 4 (IQR = 2-5) with a maximum of 10 measurements per patient. Time from ART initiation until first regimen change was considered for analysis presented in this paper. Note that models were fitted to the logarithm of CD4 counts. An example of the CD4 counts for randomly selected individuals are shown in Fig 4A. The observed individual profile with its mean plot showed an overall increase in the level of CD4 cell counts over time as shown in Fig 4B. The rise was relativity quick during the first 5 months since the start of ART and became steady after month 5. A semi-parametric mixed effect model, which takes into account both the longitudinal aspect and the subjects heterogeneity of the data was fitted. An elaborate discussion about the model and model formulation are given in the methods section. The overall trend of the data is shown in Fig 4C. In addition to the trend, the semiparametric mixed model allows the estimation of the rate of change of CD4 counts (i.e. the first derivative). A derivative equal to zero implies a constant trend with respect to time. The response of CD4 counts for ART treatment clearly observed in Fig 4D which presents the rate of CD4 change over time. Note how the derivative decreases sharply to zero in the first 10 months after the initiation of the ART treatment and thereafter remain relatively stable and closed to zero.

#### Non-Nucleotide Reverse Transcriptase Inhibitor (NNRTI) drugs

**Fig 5:**
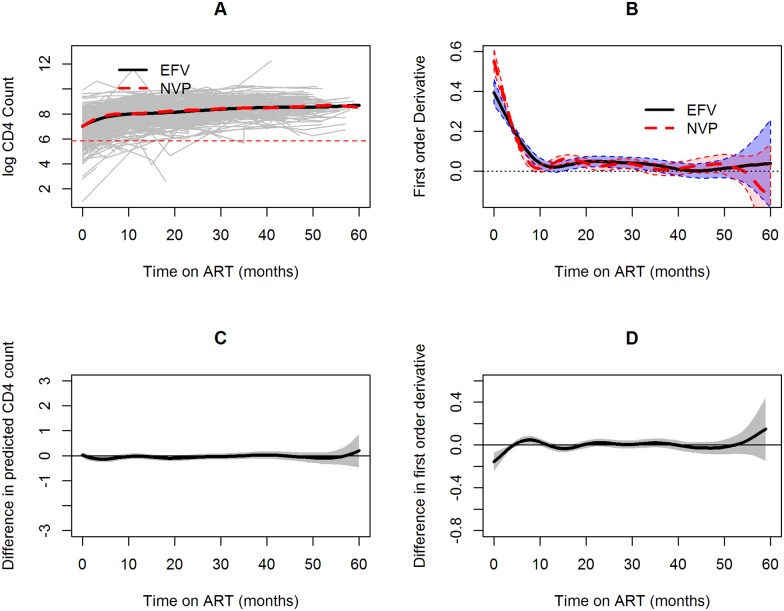
Response of log CD4 cell counts for two NNRTI drugs (efavirenz and nevirapine). (A) individual and average profiles, (B) rate of change over time for CD4 counts, (C) Estimated difference between the trend for efavirenz and nevirapine. Whenever the 95% confidence band for the curve (the gray area) covers the value of zero the difference between the two treatment is not significant.

The Semiparametric mixed effects model also allows us to compare between different treatments. Fig 5 presents a comparison between EFV and NVP. Regardless of the type of original regimen, log CD4 cell counts started increasing immediately after initiation of ART. This can be clearly seen in Fig 5A and 5B which show the predicted trend and the rate of change over time, respectively. For both treatments, a sharp increase is observed in the first 10 months after initiation of ART. Further, Fig 5C and 5D show the difference between the two treatments in both estimated log CD4 trend and the rate of change in log CD4 counts. A curve for which the 95% confidence band covers the value of zero indicates that the difference is not statistically significant. Fig 5C and 5D reveal that the response for the two NNRTI drugs seems to be differ only in the first few months after initiation of ART and thereafter the treatments are not statistically different in both CD4 count levels and the rate of change since the 95% confidence bands cover the value of zero in both cases.

#### Nucleotide Reverse Transcriptase Inhibitor (NRTI) backbone

**Fig 6:**
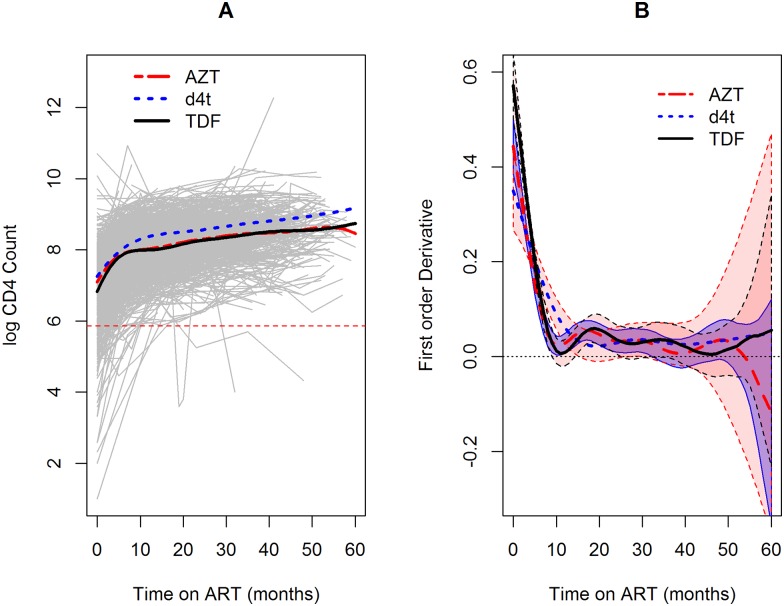
Individual and average profile plots of NRTI backbons. (A): Fitted individual and mean plots for each backbone. (B): Estimated rate of change for each backbone and 95% confidence band.

**Fig 7:**
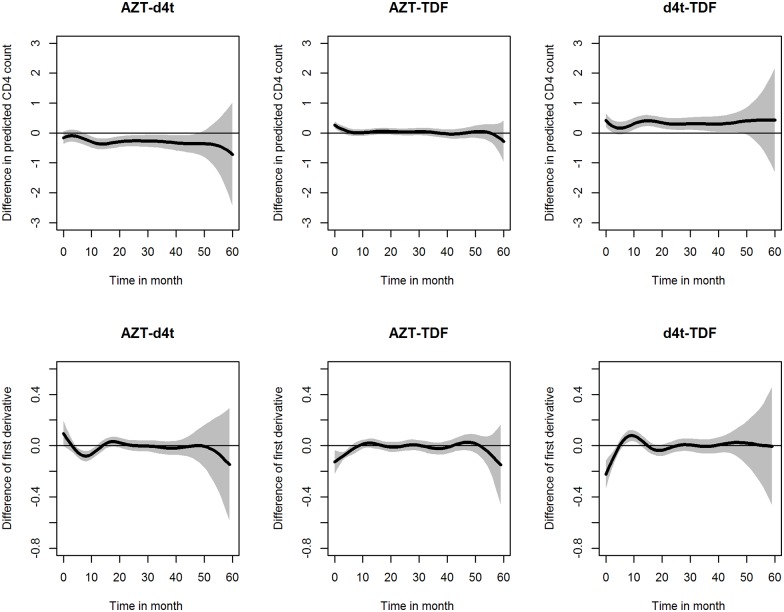
Estimated pairwise difference between the NRTI backbones. Upper panels: estimated difference between the CD4 trend over time for each pair of backbone. Lower panels: estimated difference of the rate of change of CD4 counts for each pair of backbones.

The trend of log CD4 cell counts was estimated for the three NRTI backbones (AZT, d4t and TDF). Fig 6A and the upper panels in Fig 7 show that, 10 months after the initiation of the treatment, patients treated with d4t reached higher levels of log CD4 counts compared to the patients that were treated with AZT and TDF as the backbones. Note that from 10 months after the initiation of the treatment the rate of change in log CD4 counts is the same for all backbones (see Fig 6B and the lower panels in Fig 7).

## Discussion

The Scaled-up of antiretroviral therapy has shown to be effective in improving quality of life, reducing morbidity and mortality, and increase productivity in patients infected with HIV. However, there is a need to better understand the characteristics of long-term outcomes of treatment combinations. The main aim of this study was to determine and compare the long-term response of patients on NVP or EFV based first line ART regimen in Northwest Ethiopia. The analysis presented in this paper was focused on a hospital data and the methodology presented in the paper can be applied routinely to similar dataset from other treatment centers. We have shown that treatment responses were comparable whether NVP or EFV was chosen to initiate ART for HIV-positive patients in Gondar Hospital, Ethiopia. Statistical significant difference was not detected in the risk of death or composite outcome among patients who initiated with the two NNRTI drugs after adjusting for baseline covariates. This is in agreement with other studies conducted in central Ethiopia [19], Ghana [21] and Botswana [28] which indicated a non-significant difference in the long-term effectiveness of EFV and NVP based ART regimens in the population. However, it is in contrast with findings from observational study by ART Cohort Collaboration (ART-CC) [29] and HIV-CAUSAL collaboration [17, 30] in which patients who initiated with NVP containing regimen has an increased risk of treatment failure as compared to EFV. A systematic review also revealed that EFV based first line ART is a preferred NNRTI drug in first line treatment regimen for HIV treatment as it has lower risk for treatment failure [31].

There was significant difference in the risk of composite outcomes between patients who were initiated with TDF containing ART regimen and those with AZT after controlling for NNRTI drugs. The risk on composite outcome for TDF when combined with NVP is two times higher as compared to AZT. This was supported by studies in, Zambia [22], Nigeria [32] and France [33] in which TDF containing regimen was associated with higher mortality and virologic failure. In contrast with our study, studies conducted in South African [34] and Botswana showed as TDF appeared to perform better than AZT with lower mortality. In the adjusted analysis the risk of composite outcome on TDF backbone has increased by 50% on NVP as compared to EFV indicating that TDF is more effective when administered with EFV than NVP. This is in line with finding from Thailand [35] in which the frequency of TDF-associated renal impairment was significantly higher in patients receiving TDF plus NVP compared to TDF plus EFV regimen.

This study also showed that there was a 73% increased risk of composite outcome for patients who were initiated with d4t containing regimen as compared to AZT containing regimen. The risk of composite outcome was 70% and 72% higher on d4t as compared to AZT for NVP and EFV, respectively. This was supported by a study conducted in Cameron [36] in which patients initiated with d4t has increased risk of toxicity. However, this finding was contradicted with the results reported by a study conducted in Kenya [37] in which d4t has a decreased risk of treatment failure as compared to AZT. The risk of composite outcomes was not statistically significant on d4t with any of the NNRTI drugs when lost patients were assumed censored. This indicated that the difference in risk on composite outcome on d4t versus AZT was due to lost to follow up cases. The risk of composite outcome was higher among patients who initiated ART at clinical stage 3 or 4, low CD4 count and ambulatory or bedridden functional status during ART initiation which is inline with other studies [38, 39].

Results from S3 Appendix revealed that TDF has higher risk despite the types of events as compared to AZT which is consistent with other studies [22, 33]. Although the effect of d4t and AZT on death and NNRTI modification was the same, statistical significant difference was observed on lost to follow up. Those patients who initiated with d4t containing regimen had about two fold risk of lost to follow up as compared to those who initiated with AZT containing regimen. This might be due to the long-term irreversible side effects of d4t [8]. Patients who initiated with NVP had 2.5 times higher risk of modifying the NNRTI drug than those patients who initiated with EFV. Initiating with TDF of NRTI backbone has also higher risk of NNRTI modification than those who initiated with AZT. This is in contrast with finding of the study in South Africa [40] in which rate of substitution was lower among those who initiated with TDF than AZT or d4t.

In Ethiopia, like other resource-limited countries, CD4 cell counts are used as a main surrogate marker of treatment response due to the fact that viral load monitoring is not easily accessible. We proposed a semi-parametric mixed effects model for the longitudinal evolution of CD4 cell counts in order to investigate response to treatment based on individual and average profile plots. There was an overall increase in CD4 cell counts over time which is consistent with other studies [41–44]. As shown by the first derivative plot, the rate of CD4 increase in response of treatment was high during the first 10 months and stabilized later. This analysis also revealed that there was no difference in the trend of CD4 counts in the long-run among patients who initiated with EFV or NVP containing regimen which is inline with previous study conducted in Ghana [18]. Considering only the NRTI backbones, there was difference in the evolution of log CD4 cell counts which is consistent with the study in Italy [45]. Even though d4t is less preferable by clinicians, it has better effect in improving CD4 cell counts than AZT and TDF which is supported by other study [46]. In the long run, the improvement of CD4 level was better among patients who initiated with TDF containing regimen. Even though d4t together with NVP was the combination at initiation of treatment which offer better performance in increasing CD4 cells during the first 10 months since initiation, the rate of increase was not as good as the other combinations. All options of original regimen have similar effect between month 20 and 50. The results obtained from the semi-parametric mixed effect model can be affected by the different time to lost to follow up or death reported in this paper. A joint model for CD4 counts and time to composite outcome is currently under development and will be reported in a future publication.

The main limitation of this study was the unmeasured variables effect on the findings of the study. Since the study is based on retrospective data, many covariates were not measured. Some of the variables which were not measured includes nutritional status of the patients, adherence, opportunistic infections, viral load, side effects and reasons of regimen change. This could affect the findings of this study. Another limitation was the definition of treatment failure as composite outcome which is broad. This might overestimate the event which makes comparison with other findings difficult. So, the result of this study should be interpreted with insight of these limitations.

In conclusion, the study revealed that the long-term treatment outcomes did not depend on NNRTI groups of the regimen. The outcomes of EFV containing regimen is comparable with NVP containing regimen. However, difference was observed for NRTI backbones chosen to initiate the treatment of HIV-infected patients in Ethiopia. The response of CD4 counts to treatment was high during the first 10 months and stable then after. Further clinical study is warranted in resource limited settings to investigate the effect of EFV and NVP on long-term outcomes.

## Supporting Information

S1 Appendix Statistical methods for time to event and Immunological outcomes. (PDF)

S2 Appendix Cox PH model for sensitivity analysis. (PDF)

S3 Appendix Separate analysis of long-term treatment outcomes. (PDF)

S4 Appendix Minimal dataset. (XLSX)

S1 Fig Kaplan-Meier Survival plot for all treatment regimens. (TIFF)

S2 Fig Kaplan-Meier Survival plot for selected baseline covariates. (TIFF)
