# Supplementary material for: Modeling Outcomes of First-Line Antiretroviral Therapy and Rate of CD4 Counts Change among a Cohort of HIV/AIDS Patients in Ethiopia: A Retrospective Cohort Study
Source: PLoS One. 2016 Dec 20;11(12):e0168323. doi: 10.1371/journal.pone.0168323 (PMC5173384; doi:10.1371/journal.pone.0168323)
Supplement: S1 Appendix — (PDF) [file pone.0168323.s001.pdf]

S1 Appendix.

Statistical methods for time to event outcomes

For each patient in the study we observe either the time to failure or censoring. For the censored individuals we know only the time to failure is greater than the censoring time. Denote T be a random variable representing failure time. The probability of the failure time occurring at exactly time t (out of the whole range of possible t's) can be formulated as

$$f(t) = \lim_{\Delta t \to 0} \frac{P(t \leq T < t + \Delta t)}{\Delta t},$$

where $f(t)$ is probability density function, Δt refers small change of time t. The survival function ($S(t)$) can be defined as

$$S(t) = P(T \geq t) = \int_t^\infty f(u)du.$$

Similarly, the hazard function can be defined as

$$\lambda(t) = \lim_{\Delta t \to 0} \frac{P(t \leq T < t + \Delta t | T \geq t)}{\Delta t}$$

Comparison of Kaplan-Meier survival curves between different groups was done using log rank test which is used is used to test the null hypothesis that the probability of an event occurring at any time point is the same for each group of the covariate. The test statistic is calculated as follows

$$\chi^2(log - rank) = \frac{\sum_{i=1}^k (O_i - E_i)^2}{E_i}$$

Where O_i's are the total numbers of observed events in group i, and E_i's are the total numbers of expected events in group i.

Similarly, Cox-proportional hazards model was used to check the effect of NNRTI drug, NRTI backbone and other covariates at baseline on event time. For an elaborate discussion about the log rank test we refer to [**?**]. The model can be formulated as

$$h_i(t|\mathbf{X_i}) = h_0(t)exp(\mathbf{X_i}\beta). \quad (1)$$

Where β is a $p \times 1$ vector of unknown parameters, $\mathbf{X_i}$ is the design matrix of baseline covariates such as gender, age, WHO stage, etc, and $h_0(t)$ is an unknown function giving the hazard function for the standard set of conditions $\mathbf{X_i} = 0$.

Statistical methods for Immunological outcomes

Liner mixed effects models are often used for analyzing continuous correlated data. The general liner mixed effects model can be formulated as

$$\mathbf{Y}_i(t_i) = \mathbf{X}_i\beta + \mathbf{Z}_i b_i + \varepsilon_{it_i}. \quad (2)$$

Where $\mathbf{Y}_i(t_i)(i = 1, \ldots, n_i)$ is n_i-dimensional response vector of log transformed CD4 counts for individual i at time t_i, $\mathbf{X}_i$ and $\mathbf{Z}_i$ are $n_i \times p$ and $n_i \times q$ dimensional

fixed and random effects model matrixes respectively. β_i is a p-dimensional vector of fixed effects and b_i is a q-dimensional subject specific vector of random effects.

However, many biomedical experiments generate non-linear data and imposing parametric function for the mean evolution over time might yield unsatisfactory results [**?**] [1]. In the context of HIV/AIDS data, the individual profiles are non-linear and parametric models may be too restrictive. Therefore, we propose a data-driven approach based on semi-parametric regression models. In this model, the patient-specific random intercept is used to capture correlation of the CD4 count measurement over time within the patient. We assumed patient-specific random parameters for both the linear and quadratic time effects to capture different evolution between the patients of log CD4 count over time. The Semi-parametric mixed effects model, with patient-specific random effects can be expressed as

$$\mathbf{Y}_i(t_i) = S(t_i) + b_{0i} + b_{1i}t_i + b_{2i}t_i^2 + \varepsilon_{it_i}. \tag{3}$$

here $S(t_i)$ is the non-parametric component of the model. The patient-specific random effects assumed to follow a multivariate normal distribution, $[b_{0i}, b_{1i}, b_{2i}]^T \sim MVN(\mathbf{0}, \mathbf{\Sigma}_b)$, where $\mathbf{\Sigma}_b$ denote the variance covariance matrix of patient-specific random effects. The residuals ε_{it_i} are assumed to be normally distributed with mean zero and variance σ_ϵ^2.

Here, $S(t)$ is the smoother to the log-CD4 evolution given by

$$S(t_i) = \beta_0 + \sum_{\iota=1}^{\nu} \beta_\iota f_\iota(t_i),$$

where $f_\iota(t_i)$s are a set of thin plate spline basis functions [2].

Pointwise Confidence Intervals

Let β be a parameters vector contains all fixed and random effects for the smooth terms, $\mathbf{X}_i$ is the corresponding covariates matrix, $\mathbf{Z}_i$ is the design matrix for the random effects, and $\mathbf{D}$ is the covariance matrix for the random effects. The penalized thin plate spline model can be expressed as a mixed model of the form

$$\mathbf{Y_i} = \underbrace{\mathbf{X}_i\beta_i}_{S(t)} + \mathbf{Z}_i b_i + \varepsilon_i. \tag{4}$$

For the given values of the parameters associated with the random effect and error, application of maximum likelihood and Best Prediction(BP) BLUP estimate for S is given by

$$\hat{S} = \mathbf{X}\hat{\beta}. \tag{5}$$

A point-wise confidence interval for the average fitted problem can be obtained by [3]:

$$\hat{S}(t) \pm t_{1-\alpha/2} s.d(\hat{S}(t)). \tag{6}$$

Where $s.d(\hat{S}(t))$ is the square root of the diagonal of the variance covariance matrix $\mathbf{X}\hat{V}_\beta\mathbf{X}^t$, with $\hat{V}_\beta = (\mathbf{X}^t\hat{\boldsymbol{V}}^{\mathbf{-1}}\mathbf{X} + Z)^{-1}$. Here, $\hat{V}$ is the variance and covariance matrix and Z is the wiggliness penalty matrix [**?**].

Pairwise Comparison of treatment groups

The linear mixed model formulated in (4) allows us to compare between the treatment groups in order to investigate whether there is difference between groups (comparing their average profiles). The model can be re written as

$$Y_{ig}(t_i) = \underbrace{\beta_{0g} + \beta_1 treat_g + \sum_{\iota=1}^{\nu} \beta_{g\iota} f_{\iota}(t_i)}_{S_g(t)} + b_{0i} + b_{1i} t_i + b_{2i} t_i^2 + \varepsilon_{it_i}. \tag{7}$$

where $Y_{ig}(t_i)$ is the response for the ith subject in the gth treatment group at time point t_i, $S_g(t)$ is a group specific smoother, and $f_{\iota}(t_i)$'s are a set of thin plate spline basis functions, $\beta_{\iota g}$ are the coefficients of the basis function.

We estimated different spline coefficient variances for each treatment groups g, and we used penalized thin plate regression splines with a roughness penalty on the third-order derivative $k = 3$ to obtain a smooth first order derivative. The interest is on the linear trend hypothesis test on entire profile which is stated as follows:

Let $S_g(t) = \beta_g^t F(t_i)$ be a group specific smooth curve with $F_t = [f_0(t), \dots f_{\nu}(t)]^t$, $\beta_g = [\beta_{0g}, \dots \beta_{\nu g}]^t$, and $g = 1, \dots, G$ is the group indicator. Let $S_{t_i} = [s_1(t_i), \dots, s_G(t_i)]^t$ and $\beta = [\beta_1^t, \dots, \beta_G^t]^t$. We formulate the following hypothesises;

$$H_0 : \mathbf{L} S_t = 0 \quad Vs \quad H_1 : \mathbf{L} S_t \neq 0, \tag{8}$$

which must hold for $\forall \in [t_0, t_T]$, $\mathbf{L}$ is the contrast matrix of interest for the pair of groups [4] that equation (8) is equivalent to

$$H_0 : \mathbf{L}\beta = 0 \quad Vs \quad H_1 : \mathbf{L}\beta \neq 0,$$

Where $\mathbf{L}$ is the contrast matrix of interest for the pair of groups.

The difference between groups were tested using the first derivatives. Taking the first derivative of (7) with respect to time t is given by

$$\frac{dS_g(t_i)}{dt} + b_{1i} + 2b_{2i} \times t. \tag{9}$$

Let $S_g^{'}(t_i) = \beta_g^t F^{'}(t_i)$ be the first order-derivatives of penalized thin-plate spline fit as given in (9). We can construct the point-wise confidence interval for $S_g^{'}(t_i)$ in the same fashion.

Inference about the difference between the derivative course of different group can be done in the same as describe above.

References

1. Bowman A, Azzalini A. Applied Smoothing Techniques for Data Analysis: The Kernel Approach with S Plus Illustrations. Oxford University Press; 1997.

2. Ramsay J, Silverman B. Functional Data Analysis. Second edition ed. New York: Springer; 2005.

3. Ruppert D, Wand MP, Carroll RJ. Semiparametric Regression (Cambridge Series in Statistical and Probabilistic Mathematics). Cambridge University Press; 2006.

4. Thilakarathne PJ, Clement L, Lin D, Shkedy Z, Kasim A, Talloen W, et al. The use of semiparametric mixed models to analyze PamChip peptide array data: an application to an oncology experiment. Bioinformatics. 2012;27(20):2859–2865.
