# Supplementary material for: Modeling Outcomes of First-Line Antiretroviral Therapy and Rate of CD4 Counts Change among a Cohort of HIV/AIDS Patients in Ethiopia: A Retrospective Cohort Study
Source: PLoS One. 2016 Dec 20;11(12):e0168323. doi: 10.1371/journal.pone.0168323 (PMC5173384; doi:10.1371/journal.pone.0168323)
Supplement: S2 Appendix — (PDF) [file pone.0168323.s002.pdf]

# S2 Appendix.

**Cox PH model for sensitivity analysis.** As a second sensitivity analysis, the definition of the composite outcome was modified and the new event is defined as an event whenever patients experienced either death, lost to follow up, treatment discontinuation, or NNRTI substitution. The results of the cox-regression model for the modified composite outcome are presented in Table **??**. There was no significant difference in the risk of modified composite outcome on NVP (AHR=1.02, 95%CI:0.82-1.27). TDF containing regimen at initiation has 1.73(95%CI:1.42-2.07)times higher risk on modified composite event. Baseline covariates such as sex, WHO stage, functional status and ART start year were significantly associated with composite outcomes. The risk of the modified composite outcomes on male, WHO stage III or IV, ART start year since 2010 were 1.27(94%CI:1.07-1.50), 1.35(95%CI:1.12-1.63), and 1.75(95%CI:1.43-2.13), respectively.

**Cox-regression analysis of factors associated with the modified composite outcome among HIV/AIDS patients at Gondar University Hospital, in Northwest Ethiopia, 2013**

| | Sensitivity analysis(Modified Composite outcome) | | |
|---|---|---|---|
| Covariate | Unadjusted HR(95%CI) | Adjusted HR(95%CI) | p-value |
| **Sex, n(%)** | | | |
| Female | 1 | 1 | |
| Male | 1.27(1.08-1.49) | 1.27(1.05-1.50) | 0.006 |
| **Age** | | | |
| $<$ 40 years | 1 | | |
| $\geq$ 40 years | 1.02(0.85-123) | - | - |
| **NNRTI** | | | |
| Efavirenz | 1 | 1 | |
| Nevirapine | 0.87(0.73-1.02) | 1.02(0.82-1.27) | 0.881 |
| **NRTI backbone** | | | |
| Zidovudine | 1 | 1 | |
| Stavudine | 1.33(0.98-1.81) | 1.33(0.97-1.83) | 0.072 |
| Tenofovir | 1.64(1.38-1.94) | 1.73(1.42-2.07) | <.001 |
| **WHO stage** | | | |
| I and II | 1 | 1 | |
| III and IV | 1.48(1.24-1.77) | 1.35(1.12-1.63) | 0.002 |
| **Base CD4 cells** | | | |
| <200 cells/mm3 | 1 | 1 | |
| $\geq$ 200 cells/mm3 | 0.86(0.71-1.04) | 0.95(0.78-1.15) | 0.5 |
| **Functional status** | | | |
| Ambulatory/Bedridden | 1 | 1 | |
| Working | 0.52(0.43-0.63) | 0.58(0.47-0.71) | <0.001 |
| **ART start Year** | | | |
| Before 2010 | 1 | 1 | |
| Since 2010 | 1.45(1.20-1.76) | 1.75(1.43-2.13) | <0.001 |
