# Supplementary material for: Modeling Outcomes of First-Line Antiretroviral Therapy and Rate of CD4 Counts Change among a Cohort of HIV/AIDS Patients in Ethiopia: A Retrospective Cohort Study
Source: PLoS One. 2016 Dec 20;11(12):e0168323. doi: 10.1371/journal.pone.0168323 (PMC5173384; doi:10.1371/journal.pone.0168323)
Supplement: S3 Appendix — (PDF) [file pone.0168323.s003.pdf]

S3 Appendix.

Separate analysis of long-term treatment outcomes. From the separate analysis of death, lost to follow up and NNRTI substitution (see Table below), there was no significant difference in the hazard of death and lost to follow up among patients who initiated with EFV or NVP containing regimen after adjusting for other covariates (sex, CD4 counts, WHO stage, and functional status). Though the hazard of lost to follow up was lower for patients who were initiated with NVP from bivariable analysis, it turned out to be insignificant after adjustment for other covariates. The risk of NNRTI discontinuation was 2.55(95%CI: 1.70-3.82) times higher on NVP compared to EFV after adjusting for other covariates. Initiating ART with TDF containing regimen increased the risk of death, lost to follow up and NNRTI change in reference to AZT containing backbone. The hazard of death, lost to follow up and NNRTI change increased by 1.62(95%CI: 1.23 –2.32), 1.92(95%CI: 1.40-2.64), and 1.59(95%:1.12-2.25) for patients who were initiated with TDF containing regimen compared to patients initiated with AZT, respectively. Initial ART regimen with D4T has 2.30(95%CI:1.46-3.62) times higher risk of lost to follow up compared to AZT containing initial regimen.

Cox-regression analysis of factors associated with treatment outcomes among HIV/AIDS patients at Gondar University Hospital, in Northwest Ethiopia, 2013

Outcome	Treatment	Unadjusted HR(95%CI)	P-value	Adjusted HR(95%CI)	p-value
Death	**NNRTI**				
	Efavirenz	1		1	
	Nevirapine	0.75(0.55 - 1.02)	0.065	1.14(0.81 - 1.62)	0.45
	**NRTI backbone**				
	Zidovudine	1		1	
	Stavudine	1.97(1.14 - 3.42)	0.015	1.29(0.72 – 2.29)	0.38
	Tenofovir	2.03(147 - 2.80)	<0.001	1.62(1.23 – 2.32)	0.008
Lost to follow up	**NNRTI**				
	Efavirenz	1		1	
	Nevirapine	0.59(0.45- 0.76)	<0.001	0.88(0.85- 1.20)	0.41
	**NRTI backbone**				
	Zidovudine	1		1	
	Stavudine	2.65(1.72 -4.01)	<0.001	2.30(1.46- 3.62)	0.003
	Tenofovir	2.18(1.65- 2.89)	<0.001	1.92(1.40- 2.64)	<0.001
NNRTI substitution	**NNRTI**				
	Efavirenz	1		1	
	Nevirapine	1.94(1.35-2.79)	0.003	2.55(1.70-3.82)	<0.001
	**NRTI backbone**				
	Zidovudine	1		1	
	Stavudine	0.31(0.13-0.71)	0.005	0.43(0.18-1.01)	0.053
	Tenofovir	0.84(0.62-1.16)	0.31	1.59(1.12-2.25)	0.008
