# Supplementary figures and images for: Modeling Outcomes of First-Line Antiretroviral Therapy and Rate of CD4 Counts Change among a Cohort of HIV/AIDS Patients in Ethiopia: A Retrospective Cohort Study
Source: PLoS One. 2016 Dec 20;11(12):e0168323. doi: 10.1371/journal.pone.0168323 (PMC5173384; doi:10.1371/journal.pone.0168323)

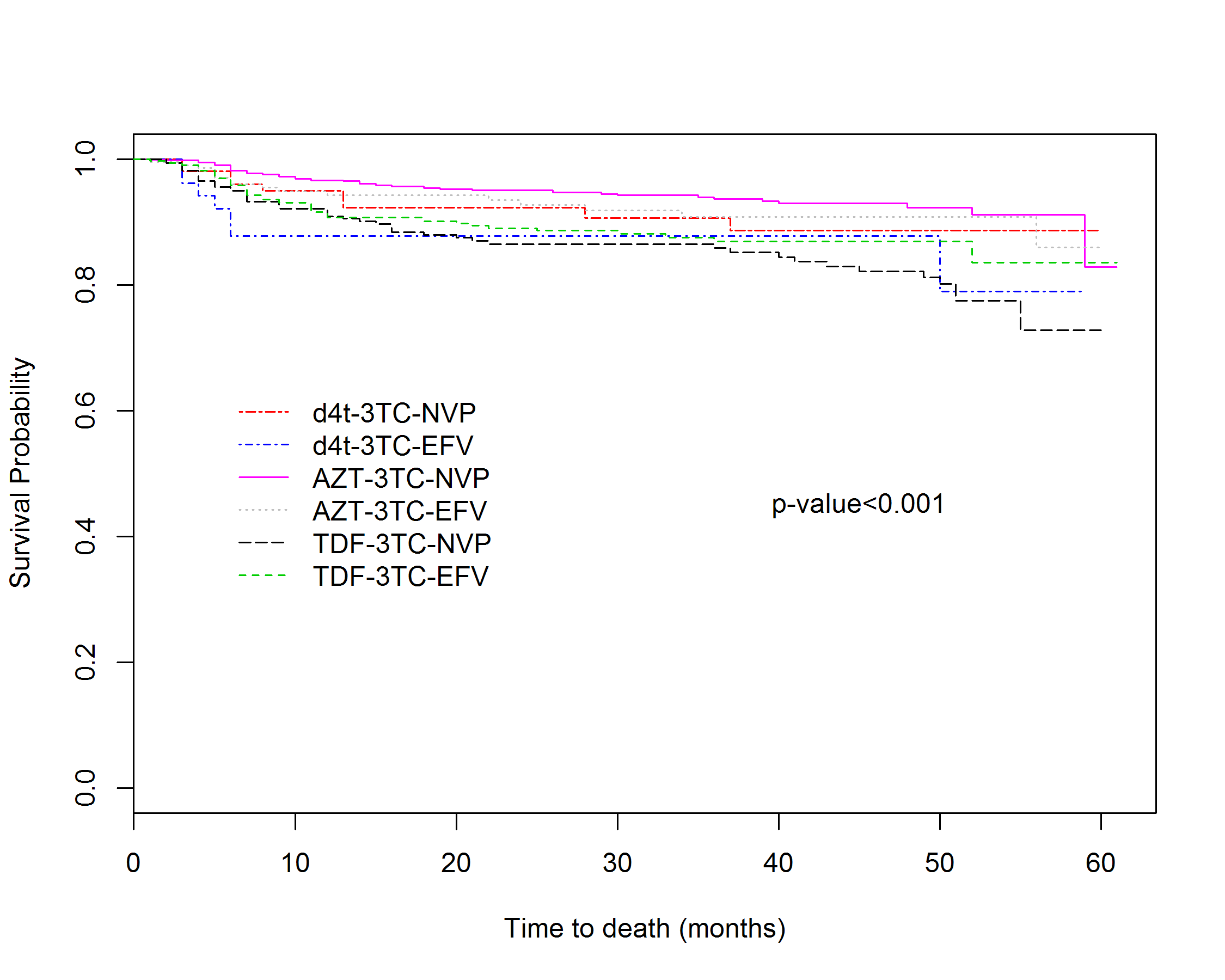

Supplement: S1 Fig — (TIFF) [file pone.0168323.s005.tiff]

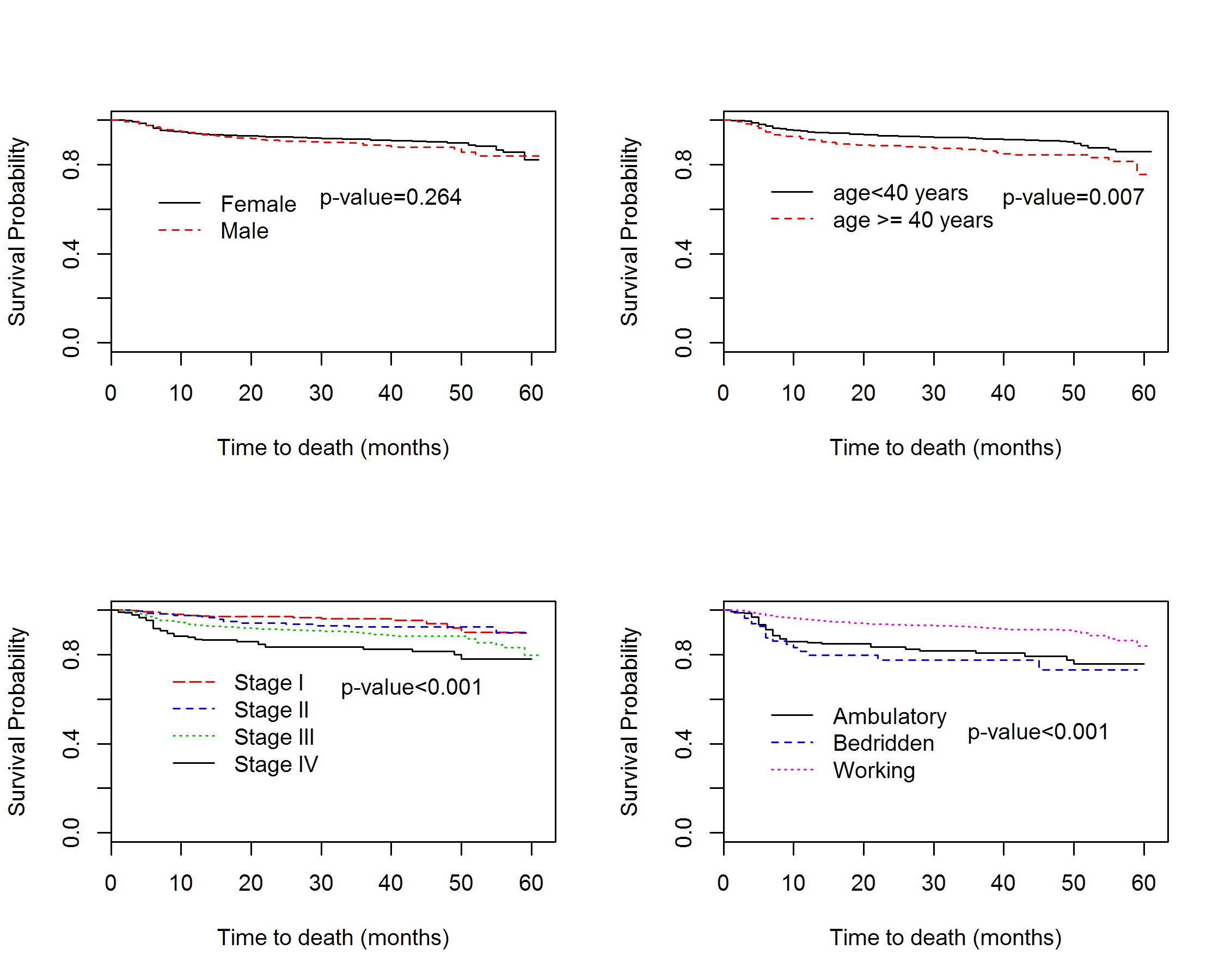

Supplement: S2 Fig — (TIFF) [file pone.0168323.s006.tiff]
